# Disease Regarded as a False or Spurious Organisation

**Published:** 1845-08

**Authors:** 


*Disease regarded as a False or Spurious Organisation.* By Dr. Jahn.—There is a good deal of German mysticism in the following remarks, which are thereby rendered rather hard to be understood in certain passages. Our readers will, however, find no difficulty in tracing out the leading features of the doctrine which the learned writer seeks to establish.—(*Rev.*)

Since the experiments of Langenbeck have become generally known, the most incredulous must surely have ceased to doubt that the propagation of (many) diseases takes place in the same manner as that of the simplest forms of animal life. The opinion of Stark, that the production,—apart from the Contagion—of diseases must be regarded as an original generation (*generatio æquivoca*,) has been much objected to ; but it seems to have been forgotten that the formation of the Infusoria, Vorticellæ, Fungi, &c., as morbid productions, occurs without contagion, and that these formations may, in the strictest sense, be regarded as instances of a veritable spontaneous generation.

A disease may justly be regarded as a *false* or *spurious* organisation ; inasmuch as, besides the normal and primitive vital type there is then present in the system a new and foreign type, which arises either from a transformation of the organic substance, or from a production of a new and intrinsic formation. This morbid type is developed and maintained at the expense of the normal life. A vast number of arguments and illustrations might be adduced to show that the *prima causa mali* in (many) diseases is really and truly of an organic or animalcular nature : let us briefly consider a few of them.

There are certain morbid productions which almost every one must regard as genuine false organisations. Of this nature are—

1. The veritable morbid formations of animal life : for example, Entozoary *anthelminths ;* various *mites*, the products of disease ; various *infusoria*, whether the product of disease, or of spontaneous developement.

2. Different vegetable productions; such as the *muscardine* of the silk-worm ; the contagious *confervæ*, discovered by Hanover in and upon the aquatic salamander ; the *fungi* found by Schoenlein existing in the patches of *porrigo lupinosa*, and which Remack has shown may be propagated by inoculation ; the various capillary *fungiform* productions observed by Fuchs and others in several exanthematous eruptions, &c. It would be difficult to admit that either these vegetable productions or their granules had entered the animal economy from without, and were not generated within. In the case of plants themselves, we have numerous instances of morbid vegetable formations, belonging to various groups of the Cryptogamic family, namely, to the *dartres*. We may mention, as belonging to this class, the *uredo*, *uromices*, *pharmegdium*, *puccinia*, *accidium*, *chrysomixa*, &c. &c.

Among the diseases of Plants, we cannot fail to recognise some under the form of *fungi*, which are generally considered as such, and are admitted into all systems of botanical arrangement : in this respect they are of the highest importance, as bearing upon the doctrine of the organic nature of diseases. The opponents of the doctrine of Spontaneous Generation will probably start the objection that the *fungi*, mentioned above as the products of disease, are never developed spontaneously in the bodies of plants themselves ; but, on the contrary, that they attach themselves and become developed in the same manner as the genuine parasitic plants that come from without. The observations, however, of *Meyer* and *Unger* on the production of the Cryptogamic formations and of the mites of the cereal grains are, we think, unrefutable, and wholly at variance with this idea.

3. Among the spontaneous animal and vegetable morbid productions, we must enumerate the *Psorosperms* described by Müller, and the corpuscles (discovered by the same naturalist) which constitute a peculiar disease of the swimming bladder of the *Gadus Callurius.* Both of these formations well deserve notice ; on the one hand, as affording a strong argument in favour of Equivocal Generation, and on the other, as having evidently a peculiar organisation, and being organised existences endowed with an individual life, and yet of such a nature as to forbid our classifying them among either plants or animals.

4. In the false or spurious organised products, we meet with formations which, on the one hand, are developed in the affected organism after the manner of its normal organs, and are associated with it ; and, on the other hand, do not belong, and are foreign or even hostile, to it, seeing that they destroy its organic matter like parasitic growths.

They are organised, and exhibit a structure like that of normal organisms ; they possess a determinate organic configuration, which often very closely resembles the form of regular or normal agents ; like the ultimate organic elements, they consist of masses which are generally of a rounded shape, and are surrounded with a proper envelope. * * * * These points have a proper vital course, proper periods, and peculiar processes of nutrition and secretion. Like many beings low in the scale of organic life, they finish by a softening and a dissolution of the mass. Moreover, they almost all possess the faculty of self-reproduction ; for, alike in the diseased and healthy organism, they can propagate (by contagion) by means of molecules, which detach themselves from the general mass, and which, being then deposited upon another point, become developed and give rise to an affection altogether similar to that from which they were derived. These molecules possess so independent a vitality that they are capable of resisting the assimilative power of the organism. It is for this reason that it is generally so difficult, and often quite impossible, to cure them ; for they possess a force of reproduction much greater than that of the organism and its different parts, being not unfrequently capable of reproducing themselves, even after they have been separated into invisible rudiments. They become fixed in the system, so that it is utterly impossible to trace any line of separation. This is clearly proved both by the morbid formations (*psorosperms* and others) discovered by Müller, by the uncertainty that still prevails as to the nature of

Acephalocysts, and by the circumstance that certain naturalists have declared that several false organisations were entozoary productions.

5. The Exanthemata are closely allied to the spurious organic productions to which we have been alluding. Contagious diseases are, like false organisations, distinguished by their generative activity and their power of self-reproduction, by their fixed periods and terms of duration, and by the co-existence of other material productions that usually accompany them.

As the entire organism is composed of primitive molecules (cellules), and as it is a universal physiological law that certain material or organic changes attend every dynamic derangement, we must attribute all morbid alterations in the body to changes in the condition of these primitive formations. It has been shown that in inflammation, typhus fever, chlorosis, scrofulous disease, scurvy and many other maladies, the globules of the blood undergo a decided change; that all sorts of external influences modify the form and colour of these globules; that in tabes dorsalis, the parts which compose the spinal marrow, and in atrophy, the molecules which compose the affected organ, are evidently more or less altered in their character; and that even in mental diseases we may often demonstrate a change in the conformation of the brain, manifested by an alteration of its primitive molecules. It is by the right application of the microscope and of chemical tests that we may hope to discover the determinate alteration in the primitive constituent elements of the organism. The production in diseases always takes place up to a certain point, independently of the idea of the individual organism; because the primitive molecules are not found in a tissue which occupies, or which has occupied, the morbidly affected point. Every disease, therefore, whatever be its name, must be considered as consisting in a false or abnormal organic production.

The researches of *Langenbeck* on the contagious nature of carcinoma, and those of *Klenck* on that of tubercles, melanosis, condylomata, warts, coryza, carbuncle, hydrophobia, and the acute exanthemata, have clearly shown that primitive, morbid and abnormal molecules may reproduce other like molecules; and, in short, that it is this very production of " molecules monstrueuses" which occurs in and constitutes the essential character of a contagious disease. The numerous examples of hereditary harelip, of supernumerary fingers and toes, and other vices of conformation, satisfactorily demonstrate that abnormal monstrous organised formations have the power of reproducing their like—a fact that quite accords with, and fully confirms, the accuracy of our position, that the morbid molecules in a living body are capable of producing molecules of a like nature to themselves.

As to the mode of developement of those vegetable and animal *protorganisatians*, which we have regarded in the preceding remarks as false organisations and morbid productions, our author suggests the following propositions to show that they are formed from an altered or vitiated state of the organic molecules and primordial *cystoblastema.*

1. It has been observed by many naturalists that, in the lowest organised forms of the animal and vegetable world, there occur transformations from one nature into another. Now the primitive molecules of the organism are endowed with a proper and independent vitality in a high degree, so that they will sometimes continue to exist when transplanted into a different organism. This vitality, we may fairly presume, becomes still greater in certain favourable circumstances. Why then, seeing that we recognise a similar augmentation of the individuality of the primitive molecules, may not external influences advance a step further, and so act that the primitive molecules or their *cystoblastema* become transformed into beings that are completely individualised, into veritable protozoites et protophytes that are susceptible of a parasitic existence, as in the above mentioned transformations of the lowest forms of organised nature? There is produced in beings of a less perfect developement the germ of other beings that are more perfect and complete. Now it is probable that a transmutation of the elementary molecules of the organism into beings possessing an individuality of existence may take place, as we have supposed. Various observations on certain animal and vegetable existences render this supposition highly probable.

2. The processes of fermentation and putrefaction are, according to the opinion of *Liebig*, movements of decomposition, in the former case of unazotised, and in the latter of azotised, substances. Both belong to what have been called chemical metamorphoses; *i. e.* to that class of phenomena in which an organic combination is decomposed by the chemical affinity of a second body, or by the influence of heat, or by some other cause, so that there are evolved from them two or more new compounds, and yet none of these elements is rendered free.

It is now ascertained that in fermenting fluids there become developed vegetable productions of an inferior order, and in putrescent animal matter certain infusoria, which, in the opinion of some distinguished naturalists, are generated by spontaneous formation; although, when once formed, they may propagate themselves by a process of vegetation. Now it is probable that not unfrequently those morbid conditions—which consist in the supervention of similar decompositions in some part of the body, in the *cystoblastema*, or in the primitive molecules of the fermentation and putrefaction—and consequently the chemical elements of the cystoblastema or of the molecules, experience a derangement in the attraction, which is indispensable to the continuance of the organization in a healthy condition: this derangement being occasioned by certain external agencies which entirely overpower the dominion of the general vital powers. Among these derangements we may enumerate various diseases, gangrenescences, putrescences, &c. But in these conditions there may possibly be developed—in consequence of the influence of external agencies, by spontaneous generation ; in other words, in consequence of the ever-acting creative power of Nature on organic matter in a state of decomposition—certain powers or properties, in the same manner as in the processes of fermentation and putrefaction. On this, as on other points, it is to the morbid states of the vegetable world that we are to look for the most conclusive instances in the way of illustration.—*Lond. Med. Chir. Review, from Haeser's Archiv. and Archives de la Medicine Belge.*

